# Neuroendocrine subtypes of small cell lung cancer differ in terms of immune microenvironment and checkpoint molecule distribution

**DOI:** 10.1002/1878-0261.12741

**Published:** 2020-07-18

**Authors:** David Dora, Christopher Rivard, Hui Yu, Paul Bunn, Kenichi Suda, Shengxiang Ren, Shivaun Lueke Pickard, Viktoria Laszlo, Tunde Harko, Zsolt Megyesfalvi, Judit Moldvay, Fred R. Hirsch, Balazs Dome, Zoltan Lohinai

**Affiliations:** ^1^ Department of Anatomy, Histology and Embryology Faculty of Medicine Semmelweis University Budapest Hungary; ^2^ Division of Medical Oncology University of Colorado Anschutz Medical Campus Aurora CO USA; ^3^ Division of Thoracic Surgery Department of Surgery Faculty of Medicine Kindai University Osaka‐Sayama Japan; ^4^ Shanghai Pulmonary Hospital Tongji University Shanghai China; ^5^ National Korányi Institute of Pulmonology Budapest Hungary; ^6^ Department of Thoracic Surgery Semmelweis University and National Institute of Oncology Budapest Hungary; ^7^ Division of Thoracic Surgery Department of Surgery Comprehensive Cancer Center Medical University of Vienna Austria; ^8^ Tisch Cancer Institute Center for Thoracic Oncology Mount Sinai Health System New York NY USA

**Keywords:** neuroendocrine, proteomics, SCLC, tumor microenvironment, tumor‐infiltrating immune cells

## Abstract

Small cell lung cancer (SCLC) has recently been subcategorized into neuroendocrine (NE)‐high and NE‐low subtypes showing ‘immune desert’ and ‘immune oasis’ phenotypes, respectively. Here, we aimed to characterize the tumor microenvironment according to immune checkpoints and NE subtypes in human SCLC tissue samples at the protein level. In this cross‐sectional study, we included 32 primary tumors and matched lymph node (LN) metastases of resected early‐stage, histologically confirmed SCLC patients, which were previously clustered into NE subtypes using NE‐associated key RNA genes. Immunohistochemistry (IHC) was performed on formalin‐fixed paraffin‐embedded TMAs with antibodies against CD45, CD3, CD8, MHCII, TIM3, immune checkpoint poliovirus receptor (PVR), and indoleamine 2,3‐dioxygenase (IDO). The stroma was significantly more infiltrated by immune cells both in primary tumors and in LN metastases compared to tumor nests. Immune cell (CD45^+^ cell) density was significantly higher in tumor nests (*P* = 0.019), with increased CD8^+^ effector T‐cell infiltration (*P* = 0.003) in NE‐low vs NE‐high tumors. The expression of IDO was confirmed on stromal and endothelial cells and was positively correlated with higher immune cell density both in primary tumors and in LN metastases, regardless of the NE pattern. Expression of IDO and PVR in tumor nests was significantly higher in NE‐low primary tumors (vs NE‐high, *P* < 0.05). We also found significantly higher MHC II expression by malignant cells in NE‐low (vs NE‐high, *P* = 0.004) tumors. TIM3 expression was significantly increased in NE‐low (vs NE‐high, *P* < 0.05) tumors and in LN metastases (vs primary tumors, *P* < 0.05). To our knowledge, this is the first human study that demonstrates *in situ* that NE‐low SCLCs are associated with increased immune cell infiltration compared to NE‐high tumors. PVR, IDO, MHCII, and TIM3 are emerging checkpoints in SCLC, with increased expression in the NE‐low subtype, providing key insight for further prospective studies on potential biomarkers and targets for SCLC immunotherapies.

## Graphical abstract

Small cell lung cancer (SCLC) has recently been subcategorized into neuroendocrine (NE)‐high and NE‐low subtypes. This study demonstrates that NE‐low SCLCs are associated with increased immune‐cell infiltration compared to NE‐high tumours. We found expression of PVR, indoleamine 2,3‐dioxygenase, MHCII, and TIM3 to be increased in the NE‐low subtype, highlighting these molecules as potential biomarkers and targets for SCLC immunotherapies.

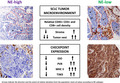

**Abbreviations**
APC: antigen‐presenting cell
DC: dendritic cell
FFPE: formalin‐fixed paraffin‐embedded
IDO: indoleamine 2,3‐dioxygenase
LN: lymph node
MHC II: major histocompatibility complex II
NE: neuroendocrine
NK: natural killer
OS: overall survival
PFS: progression‐free survival
PVR: poliovirus receptor
SCLC: small cell lung cancer
TIL: tumor‐infiltrating lymphocytes
TIM3: T‐cell immunoglobulin and mucin domain‐containing 3
TMA: tissue microarray

## 1 Introduction

Very recently, substantial milestones have been achieved in the understanding of small cell lung cancer (SCLC) biology. Two recent randomized trials comparing etoposide‐platinum doublet therapy alone to the same therapy plus a checkpoint inhibitor (atezolizumab or durvalumab) as first‐line therapy showed significant increases in progression‐free survival (PFS; 4.3–5.2 month), response rate, and overall survival (OS; 12.3–13 vs 10.3 months) with the immunotherapy [1, 2]. However, these benefits are limited, and biomarkers, such as smoking status, tumor mutation burden (TMB), and programmed cell death‐ligand 1 (PD‐L1) expression, did not predict outcome. The lack of a biomarker and the limited benefit for a small portion of patients points toward the idea that SCLC might be associated with a different immunological microenvironment [3, 4]. Furthermore, a lack of tumor tissue availability due to disease aggressiveness limits our understanding of crucial immunological mechanisms, including immune cell infiltration, intertumor and intratumor heterogeneity, and is one reason behind the long‐term failure of immunotherapies. Moreover, in many patients, lymph node (LN) metastases are the primary motivators for rapid disease progression, and their immunological environment is far less understood.

Small cell lung cancer is no longer considered as a single‐disease entity, and subtypes are defined by distinct RNA gene expression profiles which can be classified into neuroendocrine (NE)‐high and NE‐low tumors, which may have different immunogenicity [5]. NE‐high is characterized by decreased immune cell infiltration defined as a cold or ‘immune desert’ phenotype, based on low levels of immune cell‐related RNA expression. In contrast, NE‐low was associated with tumors with increased immunogenicity, in other words ‘hot’ or ‘immune oasis’ phenotype [5, 6, 7, 8]. Consequently, NE‐low SCLC patients may more likely respond to immunotherapies [9, 10]. The immune infiltrate is comprised of innate and adaptive immune cells, whose populations are heterogeneous across tumor types and patients and include nonspecific immune cell types, such as macrophages, neutrophil granulocytes, dendritic, mast and natural killer (NK) cells, or effector cells of specific immunity, like B‐ and CD3^+^ T cells (CD4^+^ T helper, CD8^+^ cytotoxic T, and regulatory T [Treg] cells), localized in tumor nests, or adjacent tumor stroma [11]. A high number of dendritic cells (DCs), NK cells, B cells, and CD8^+^ T cells were associated with improved prognosis, while the presence of Treg cells correlates with decreased survival time in NSCLC [12, 13]. The invasion of tumor nests by immune cells confers better OS in lung cancer and other malignancies [14, 15].

In addition to the presence of tumor‐infiltrating immune cells, the expression of specific immune checkpoints is also a crucial immune‐suppressing factor in many cancers. Poliovirus receptor (PVR), an important factor in the SCLC microenvironment, is an adhesion molecule involved in cell motility, as well as NK cell and T‐cell‐mediated immunity. PVR is relatively absent in normal tissues, but regularly overexpressed in malignancies promoting tumor cell invasion and migration [16]. PVR expression was detected at low levels in multiple cell types of epithelial origin and overexpressed in cancers of epithelial and neural origins [17, 18, 19]. PVR was also proved to play a crucial role in oncoimmunity, as a ligand of coinhibitory receptor TIGIT and CD96 on NK and T cells [20]. Recently, it was reported that PVR is highly expressed in SCLC cell lines with minimal expression observed on immune cells in the tumor microenvironment [21]. Indoleamine‐2,3‐dioxygenase 1 (IDO) is a key factor in defining cancer immunogenicity [22] and is a cytosolic enzyme catalyzing the first and rate‐limiting step of tryptophan (Trp) catabolism. Multiple studies revealed that the accumulation of Trp metabolites promotes the differentiation of Treg cells and induces the apoptosis of effector T cells with consequent immunosuppression [23, 24]. IDO is overexpressed in many tumor types exploiting immunosuppressive mechanisms to promote their spread and survival [25].

While antigen‐presenting cells (APCs) constitutively express major histocompatibility complex (MHC) class II; many other cell types, including malignant cells, are also capable of expressing MHC II [26]. Tumor‐specific expression of the MHC II molecule was shown to increase tumor recognition by immune cells and consequently may play a pivotal role in immunotherapy [27]. Of note, MHC II expression by tumor cells has been associated with improved prognosis and response to immunotherapy in breast cancer [28] and melanoma [29]. Lymphocyte exhaustion is a common cause of anergy in antitumor immune responses [30]. TIM‐3, also known as HAVCR2, is a negative regulatory immune checkpoint and is detected in different types of immune cells, including T and B cells, macrophages, DCs, NK, and mast cells [31]. Its negative role in anticancer immunity was shown in mediating T‐cell exhaustion [32, 33], where T‐cell immunoglobulin and mucin domain‐containing protein 3 (TIM3)^+^ CD8^+^ T cells exhibited impaired Stat5 and p38 signaling.

This study focuses on the evaluation and quantification of immune cell infiltration by localization and distribution patterns in the stroma and tumor nests according to SCLC NE subtypes. In addition, SCLC tumors were evaluated for the expression of MHC II, emerging immune checkpoints PVR, IDO, and lymphocyte exhaustion markers, including TIM3 to allow for new trials of immune therapy in these SCLC subsets.

## 2 Materials and methods

### 2.1 Ethics statement

Research was conducted in accordance with the guidelines of the Helsinki Declaration of the World Medical Association. The approval of the Hungarian Scientific and Research Ethics Committee of the Medical Research Council, (ETTTUKEB‐7214‐1/2016/EKU) was obtained and waived the need for individual informed consent for this study. After the collection of clinical data, patient identifiers were removed so that patients may not be identified either directly or indirectly.

### 2.2 Study population

A total of 32 histologically confirmed early‐stage SCLC patients with available primary tumor tissue and matched LN metastases were included in our study as previously described [34]. All patients underwent surgical resection in the period from 1978 to 2013 at the National Koranyi Institute of Pulmonology. Formalin‐fixed, paraffin‐embedded (FFPE) tissue samples from primary tumors and LN metastases were obtained at the time of lung resection surgery. Clinicopathological characteristics were described earlier [34].

### 2.3 Tissue processing

Small cell lung cancer patient tumors were obtained by surgical resection and were fixed and processed into paraffin blocks. Tissue microarray (TMA) construction from FFPE blocks was performed as previously described [35]. Briefly, 4‐micron sections from each tissue block were prepared using a HM‐315 microtome (Microm, Boise, ID, USA) and placed on charged glass slides (Colorfrost Plus, #22‐230‐890; Fisher, Racine, WI, USA). Slides were stained for H&E on an automated Tissue‐Tek Prisma staining platform (Sukura, Osaka, Japan). H&E slides were reviewed by a laboratory pathologist for tumor area and the tumor border marked. Marked‐stained sections were used to guide the technician as to the location for punch tissue removal. Two 1‐mm punches of tissue were taken from each donor tissue block for primary tumors, and one 1‐mm punch from LN metastases blocks and seated into a recipient paraffin block in a positionally encoded array format (MP10 1.0 mm tissue punch on a manual TMA instrument; Beecher Instruments, Sun Prairie, WI, USA).

### 2.4 Molecular analysis

RNA expression data from primary and LN FFPE tumor tissue samples were obtained using the HTG EdgeSeq Targeted Oncology Biomarker Panel as previously described [34]. Tumors were clustered into NE‐low (*n* = 21) and NE‐high (*n* = 43) subtypes according to their NE gene expression patterns as previously reported [34].

### 2.5 Immunohistochemistry

Four‐micron‐thick sections were cut from FFPE TMA blocks for IHC staining. Slides were stained on a Leica Bond RX autostainer using rabbit monoclonal antibody for IDO (#86630), CD45 (#13917), CD3 (#85061), CD8 (#8112), MHC II (#68258), PD‐L1 (13684S), and PVR (#81254) from Cell Signaling (Danvers, MA, USA) and diluted 1 : 200 with Cell Signaling antibody diluent (#8112) prior to staining. Antibodies TIM3 (PA0360), LAG3 (PA0300), and PD‐1 (PA0216) were from Leica Biosystems (Wetzlar, Germany) diluted 1 : 200 with Leica antibody diluent. Slides were stained using the Bond Polymer Refine Detection kit (#DS9800) with Leica IHC Protocol F and exposed to epitope retrieval 1 (low pH) for 20 min. Following staining, slides were cleared and dehydrated on an automated Tissue‐Tek Prisma platform and cover‐slipped using a Tissue‐Tek Film cover slipper. The detection of protein expression was optimized in human tonsil and thymus tissue as a positive control.

### 2.6 Cell counting and morphometry

Images of TMA sections were captured *via* a BX53 upright Olympus microscope and a DP74 color CMOS camera with 10× magnification objectives in 20MP resolution for scoring and cell counting and with 20× magnification for representative images from tumor tissues. Morphometry based on stromal and tumor nest area measurements was performed by olympus cellsens dimensions Software package by manual annotation of measured areas, as previously described [36]. In the case of primary tumors, for one patient, two different TMA specimens were analyzed (A and B), retrieved from different regions of resected tumors. In the case of LN metastases, one TMA specimen was prepared from each LN sample. From all TMA blocks, two separate four‐micron‐thick sections (with a minimum of 100‐μm distance in *Z* between them) were quantified using high resolution (20MP) 10× magnification images. Positive cells for immune markers CD45, CD3, CD8, IDO, and TIM3 were identified by the presence of brown DAB precipitation around hematoxylin‐stained cell nuclei by a systematic quantitative method based on software‐assisted, manual cell counting by two independent observers using the cell counter plug‐in of imagej software [37]. PVR and MHCII expression was assessed semiquantitatively, where 0 = negative, 1 = low, 2 = moderate, 3 = strong, 4 = very strong expression scores were given for each specimen. Immune cells and tumor cells regarding MHC II—positivity were identified according to nuclear and cellular morphology. Quantification of IDO and TIM3 expression was based on positive cell numbers in stroma and tumor nests in the whole visual field (10× magnification) of two separate sections of one TMA core. No DAB signs without the characteristic cellular shape or without the co‐presence of nuclear staining were included in the calculations. Stromal and tumor nest total areas were measured using the area measurement tool in the olympus cellsens dimensions software package. Square micrometers (μm^2^) were converted to square millimeters (mm^2^) for calculation of cell density parameters in statistical analyses. Regions of apoptosis, necrosis, and damage or disruptions in the sections were not included in the measurements. Results (cell numbers and areas) from separate sections of the same TMA punches were averaged before statistical assessment.

### 2.7 Statistical methods

First, we used the Kolmogorov–Smirnov test to determine which variable follows a normal distribution, where CD45, CD3, CD8, IDO, PVR, TIM3, and MHC II do not, but CD3/CD45 and CD8/CD3 cell density ratios followed a normal distribution. Next, we used the Wilcoxon matched‐pairs signed ranks test to test whether core A and B population mean rank differ. However, we found no significant differences regarding any variables. Accordingly, we used average core A and B values in further statistical analyses. We used the Mann–Whitney *U*‐test to compare CD45, CD3, CD8, IDO, and TIM3 expressions between primary tumors and LN metastases and between NE‐low and NE‐high subtypes in the stroma or tumor compartments. To compare NE‐low and NE‐high subtypes in the case of ordinal variables, PVR and MHCII, we used Mann–Whitney *U*‐test. *P*‐values < 0.05 indicate the significance and all *P*‐values were two‐sided. We found significant differences for all variables between tumor core A and stroma core A, or between tumor core B and stroma core B (Wilcoxon matched‐pairs signed ranks test). Accordingly, Wilcoxon matched‐pair test was further used to compare CD45, CD3, and CD8 expression between stroma and tumor nests in primary tumors or LN metastases. We used unpaired Student's *t*‐test to analyze variables with normal distribution. Spearman's rank correlation was used for continuous variables such as CD45, CD3, CD8, and IDO and Kendall's Tau‐b (Kendall rank correlation coefficient) for ordinal variable, PVR. The correlation coefficient (*r*) can vary between −1 and 1. We define no correlation (0 < *r* < 0.2), weak positive correlation (0.2 < *r* < 0.4), moderate positive correlation (0.4 < *r* < 0.6), and strong positive correlation (0.6 < *r* < 1). All statistical analyses were implemented using the pasw statistics 22.0 package (SPSS Inc., Chicago, IL, USA).

## 3 Results

**Fig. 1:**
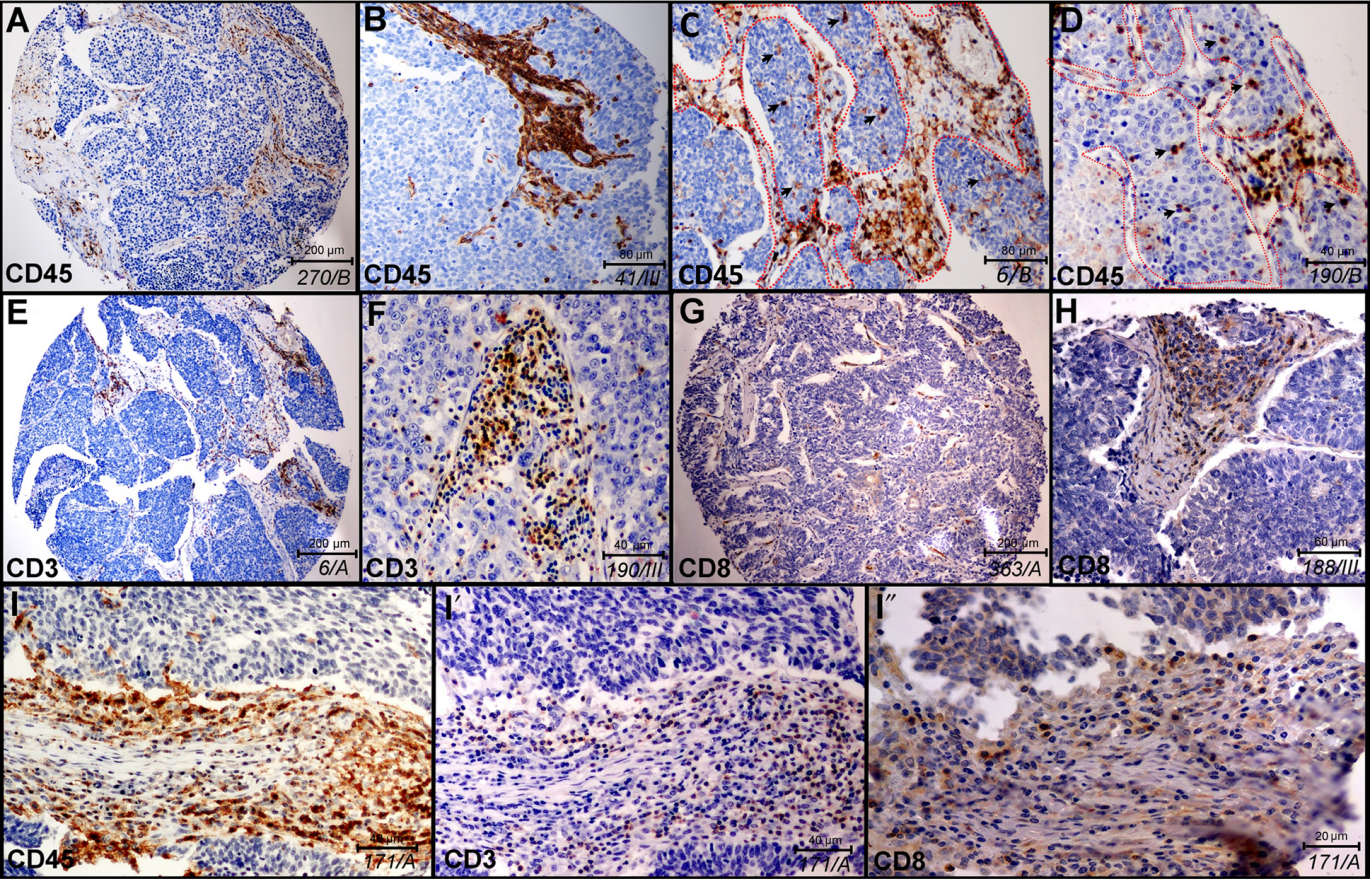
Histological localization of major immune cells in SCLC in representative tissue samples. Qualitative *in situ* IHC data on the histological distribution of immune cells show high immune cell density in the stroma and a low number of labeled cells in tumor nests (A, B magnified image) stained with anti‐CD45 antibody and hematoxylin (ID of samples in italics). Infiltration of CD45^+^ immune cells in tumor nests can be low (A, B) or moderate (C, D), where dashed line signs the border of stroma and epithelial tumor nests (C, D) and arrowheads show immune cells inside tumor nests (D). Sections of whole TMA specimens stained with anti‐CD3 and anti‐CD8 antibodies show the presence of CD3^+^ T cells (E, F) and CD8^+^ cytotoxic T cells (G, H) in low (E, G) and high (F, H) magnification images in tumor stroma and sparsely in tumor nests. High magnification images of consecutive sections from the same TMA specimen and region of interest show CD45 (I), CD3 (I′) and CD8 (I″) labeling of tumor‐infiltrating immune cells.

In our study, we aimed to reveal the precise distribution pattern of immune cells *in situ* on SCLC tissue samples. For this, we performed IHC on serial sections of FFPE TMA samples and demarcated the histological compartments of tumor stroma (stroma) and epithelial tumor nests (tumor) with consequent software‐aided area measurement, followed by cell counting in every sample. First, we analyzed the histological distribution of immune cells in stroma vs tumor nests in representative samples shown in Fig. 1. CD45 immunolabeling identifies a high number of immune cells in the stroma (Fig 1A,B), but a limited number of cells in epithelial tumor nests (Fig. 1C,D). Borders of fibrous stromal strands and tumor nests are shown with dashed lines, and immune cells inside tumor nests are indicated with arrowheads in Fig. 1C,D on representative TMA sections. CD3 labels all mature T‐cell populations of round cellular morphology (Fig. 1E,F), whereas CD8 represents the general marker for cytotoxic (effector) T cells (Fig. 1G,H). Successive sections from the same primary tumor sample of SCLC patient show the expression of CD45 (Fig. 1I), CD3 (Fig. 1I′) and CD8 (Fig 1I″) on consecutively narrower cell populations (immune cells, T cells, CD8^+^ T cells) in the same area of the TMA specimen. Based on our *in situ* HE‐stained sections, the stroma and tumor area ratio were similar in primary tumors and LN metastases (Fig. S1A), and there were no statistically significant differences according to NE subtypes (Fig. S1B).

### 3.1 Immune cell distribution in primary tumors and lymph node metastases

**Fig. 2:**
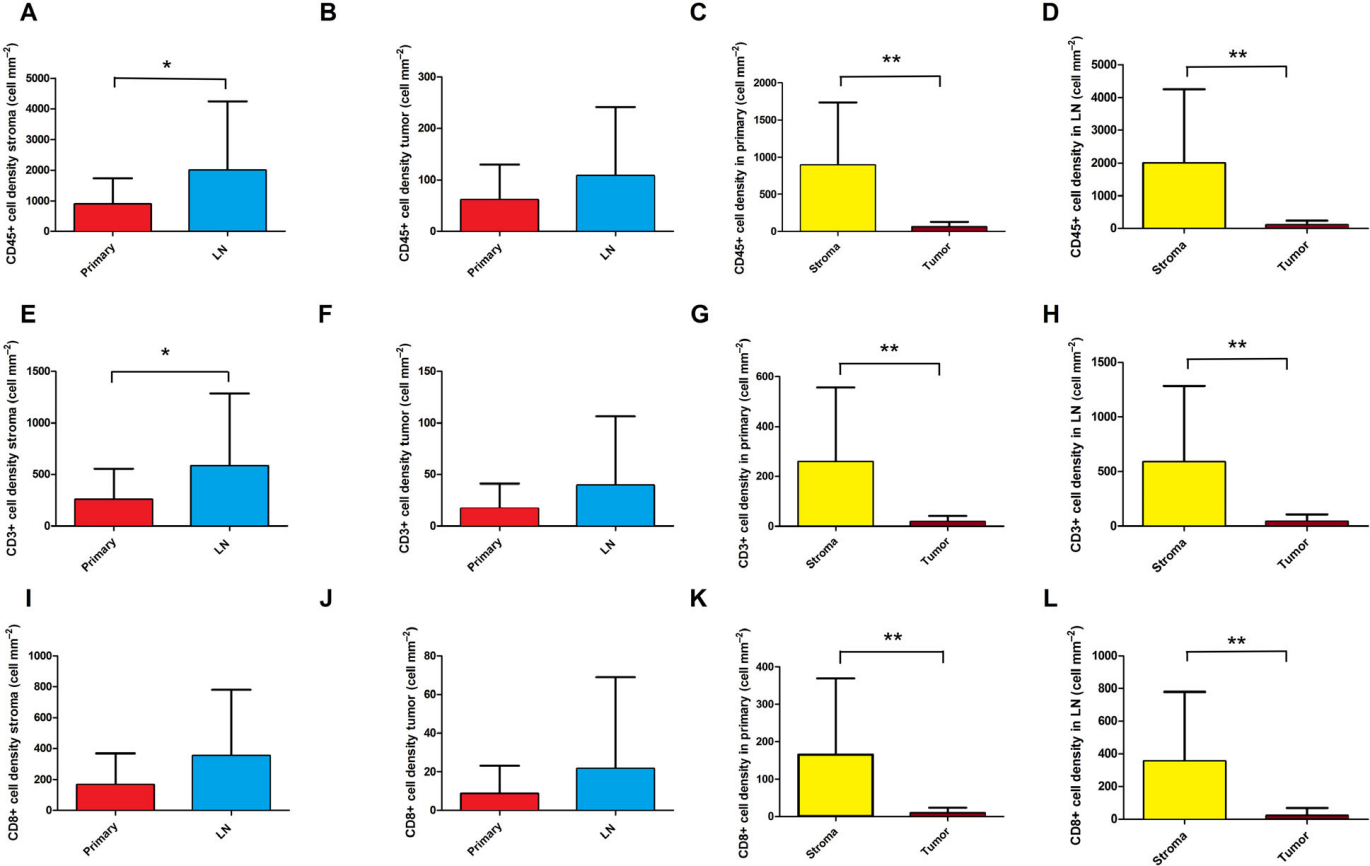
Immune cell distribution in primary SCLC tumors and matched LN metastases according to stroma and tumor nests. Significantly higher cell density was determined in the stroma of LN metastases compared to primary tumors, for CD45 (893.1 ± 159.4 vs 1993 ± 426.6 cell·mm^−2^, *P* = 0.049, *n* = 59, A), CD3 (258.5 ± 56.47 vs 585.4 ± 132.2 cell·mm^−2^, *P* = 0.033, *n* = 56, E), but not for CD8 (165.9 ± 38.46 vs 356.0 ± 80.07 cell·mm^−2^, *P* = 0.075, *n* = 58, I). Immune cell density showed no significant difference in tumor nests (tumor) in LN metastases compared to primary tumors, for CD45 (61.52 ± 13.21 vs 107.9 ± 26.25 cell·mm^−2^, *P* = 0,215,, *n* = 59, B), CD3 (17.13 ± 4.62 vs 39.50 ± 13.13 cell·mm^−2^, *P* = 0.251, *n* = 56, F), and CD8 (8.66 ± 2.78 vs 21.62 ± 9.295 cell·mm^−2^, *P* = 0.332, *n* = 58, J). Moreover, the stroma of primary tumors are significantly more infiltrated by major immune cells vs tumor nests, for CD45 (893.1 ± 159.4 vs 61.52 ± 13.21 cell·mm^−2^, *P* < 0.001, *n* = 31, C), CD3 (258.5 ± 56.47 vs 17.13 ± 4.62 cell·mm^−2^, *P* < 0.001, *n* = 29, G), and CD8 (165.9 ± 38.46 vs 8.67 ± 2.78 cell·mm^−2^, *P* < 0.001, *n* = 30, K). The stroma of LN metastases are significantly more infiltrated by major immune cells vs tumor nests, for CD45 (1993 ± 426.6 vs 107.9 ± 26.25 cell·mm^−2^, *P* < 0.001, *n* = 28, D), CD3 (585.4 ± 132.2 vs 39.50 ± 13.13 cell·mm^−2^, *P* = 0.002, *n* = 26, H), and CD8 (356.0 ± 80.07 vs 21.62 ± 9.295 cell·mm^−2^, *P* < 0.001, *n* = 28, L). Wilcoxon matched‐pair test was used to compare immune cell densities in the stromal vs intratumoral compartments. Mann–Whitney *U*‐test was used to compare immune cell densities in the stroma and tumor compartments of primary tumors vs LN metastases. Metric data were shown as mean and corresponding SEM, and graphs indicate the mean and corresponding 95% CI. Statistical significance *P < 0.05; **P < 0.01.

Next, we compared the presence of immune cells according to anatomic localization. Immune cell marker expression according to primary tumors vs LN metastases is shown in Fig. 2. We found that CD45^+^ (Fig. 2A,B), CD3^+^ (Fig. 2E,F), and CD8^+^ (Fig. 2I,J) immune cell density was significantly higher in the stroma of LN metastases compared to primary tumors, but there was no significant difference in the case of tumor nests (tumor). Moreover, the stroma of primary tumors were significantly more infiltrated by major immune cells vs tumor nests, in primary tumors (Fig. 2,G,K; *P* < 0,001) and in LN metastases (Fig. 2D,H,L; *P* < 0,001). Figure S2 shows the relative distribution of major immune cells in stroma vs tumor nests, according to primary tumors and LN metastases.

### 3.2 Immune cell distribution according to NE subtypes and tumor compartments

**Fig. 3:**
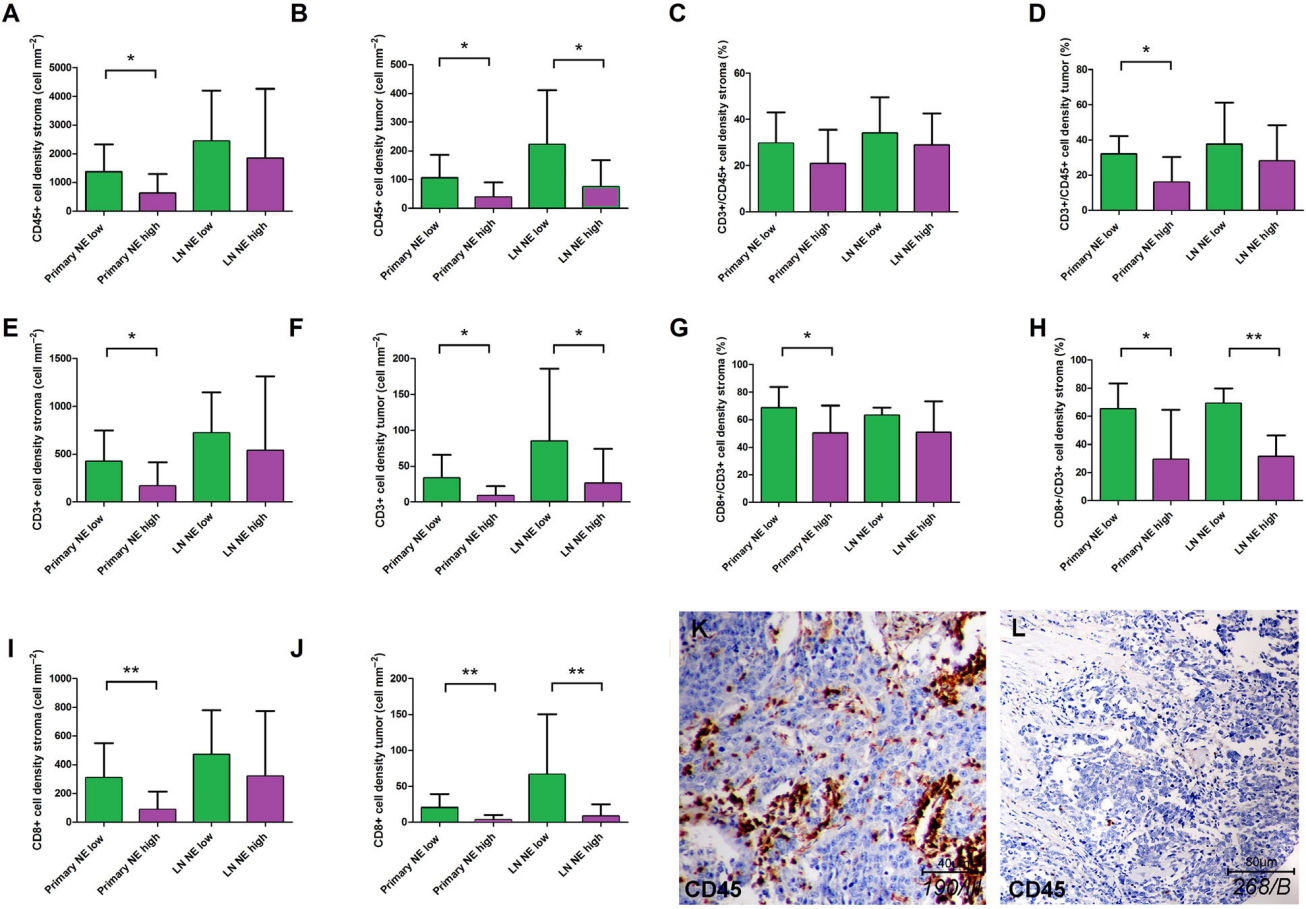
Immune cell distribution in primary SCLC tumors and matched LN metastases according to stroma and tumor nests based on NE tumor subtypes. Stained specimens revealed increased CD45^+^ cell densities in NE‐low primary tumor subtypes compared to NE‐high ones including both stroma (1371 ± 300.5 vs 627.5 ± 156.8 cell·mm^−2^, respectively, *P* = 0.02, *n* = 31, A) and tumor nests (tumor; 106.2 ± 26,67 vs 39,17 ± 12.04 cell·mm^−2^, respectively, *P* = 0.019, *n* = 31, B). We found a significantly increased density of CD45^+^ cells in NE‐low LN metastases compared to NE‐high subtypes in tumor nests (221.0 ± 7.06 vs 73.95 ± 20.86 cell·mm^−2^, respectively, *P* = 0.035, *n* = 28, B) but not in stroma (2436 ± 668.1 vs 1845 ± 527.7 cell·mm^−2^, respectively, *P* = 0.071, *n* = 28, A). There were significantly increased densities of CD3^+^ cells in NE‐low primary tumor compared to NE‐high subtypes in stroma (423.7 ± 103 vs 166.8 ± 58.09 cell·mm^−2^, respectively, *P* = 0.022, *n* = 29, E) and in tumor nests (33.22 ± 10.89 vs 9.08 ± 3.11 cell·mm^−2^, respectively, *P* = 0.035, *n* = 29, F). We found a significantly increased density of CD3^+^ cells in NE‐low LN metastases compared to NE‐high subtypes in tumor nests (84.67 ± 41.47 vs 25.95 ± 10.83, *P* = 0.032, *n* = 26, F) but not in stroma (721.9 ± 160.4 vs 539.9 ± 168.8, *P* = 0.527, *n* = 26, E). There were significantly increased densities of CD8^+^ cells in NE‐low primary tumor compared to NE‐high subtypes both in stroma (307.5 ± 77.11 vs 87.28 ± 29.77 cell·mm^−2^, *P* = 0.006, *n* = 30, I) and in tumor nests (20.56 ± 6.11 vs 2.72 ± 1.66, *P* = 0.003, *n* = 30, J). We found a significantly increased density of CD8^+^ cells in NE‐low LN metastases compared to NE‐high subtypes in tumor nests (66.17 ± 34.30 vs 8.25 ± 3.76 cell·mm^−2^, respectively, *P* = 0.006, *n* = 28, J) but not in stroma (469.4 ± 117.6 vs 318.2 ± 99.36 cell·mm^−2^, respectively, *P* = 0.063, *n* = 28, I). According to NE‐low and NE‐high primary tumors the CD3^+^/CD45^+^ cell ratio was limited to 29.55 ± 2.25 % and 20.78 ± 3.67% (*P* = 0.14) in the stroma, and 32.07 ± 3.84% and 15.86 ± 3.88% (*P* = 0.016) in tumor nests (C, D). According to NE‐low and NE‐high primary tumors, the CD8^+^/CD3^+^ cell ratio was limited to 68.6 ± 4.84% and 50.33 ± 5.14% (*P* = 0.022) in the stroma and 65.21 ± 6.79% and 29.14 ± 13.4% (*P* = 0.033) in tumor nests, respectively (G, H). According to NE‐low and NE‐high LN metastases the CD8^+^/CD3^+^ cell ratio was limited to 63.14 ± 2.14% and 50.89 ± 5.11% (*P* = 0.16) in the stroma and 69 ± 4.41% and 31.27 ± 4.54%, *P* < 0.001 in tumor nests, respectively (G, H). CD45 immunolabeling on a representative section of NE‐low (K) LN metastasis shows highly infiltrated stroma and tumor nests, whereas tumor‐infiltrating immune cells are absent both in the stroma and in the tumor nests on the sample of NE‐high primary tumor (L). Mann–Whitney U‐test was used to compare immune cell densities in the stroma and tumor compartments in NE‐high vs NE‐low primary tumors and LN metastases. Student's *t*‐test was used to compare CD3/CD45 and CD8/CD3 cell density ratios in NE‐high vs NE‐low primary tumors and LN metastases. Metric data were shown as mean and corresponding SEM, and graphs indicate the mean and corresponding 95% CI. Statistical significance *P < 0.05; **P < 0.01.

Table S1 shows the key tumor microenvironmental protein expression data according to NE‐low vs NE‐high SCLC subtypes. In primary tumors, we found a significantly increased stromal density of CD45^+^ cells (Fig. 3A; *P* = 0.02), CD3^+^ cells (Fig. 3E; *P* = 0.022), and CD8^+^ cells (Fig. 3I; *P* = 0.006) in NE‐low compared to NE‐high subtypes. Similarly, there were significantly increased cell densities of CD45^+^ cells (Fig. 3B; *P* = 0.019), CD3^+^ cells (Fig. 3F; *P* = 0.035), and CD8^+^ cells (Fig. 3J; *P* = 0.003) in tumor nests as well. Next, we analyzed LN metastases in terms of NE subtypes and immune cell distribution, where we found a significantly increased density of CD45^+^, CD3^+^, and CD8^+^ cells in NE‐low compared to NE‐high LN metastases in tumor nests (Fig. 3B,F,J), but not in the stroma (Fig. 3A,E,I). Figure 3C,D,G,H show the relative immune cell distributions according to NE subtypes, where CD3/CD45 and CD8/CD3 ratios were significantly increased in NE‐low (vs NE‐high), tumors (*P* < 0.05) in tumor nests, but not in stroma. Figure 3K shows a representative sample of NE‐low SCLC subtype stained with CD45, where massive infiltration of stroma and a relatively high number of immune cells in tumor nests are characteristic. On the contrary, a typical ‘immune desert’ or infiltrate‐excluded phenotype with scattered CD45^+^ cells both in stroma and in tumor nests is shown in Fig. 3L from a representative sample of NE‐high SCLC tumor subtype.

### 3.3 Immune checkpoint expression and NE subtypes

**Fig. 4:**
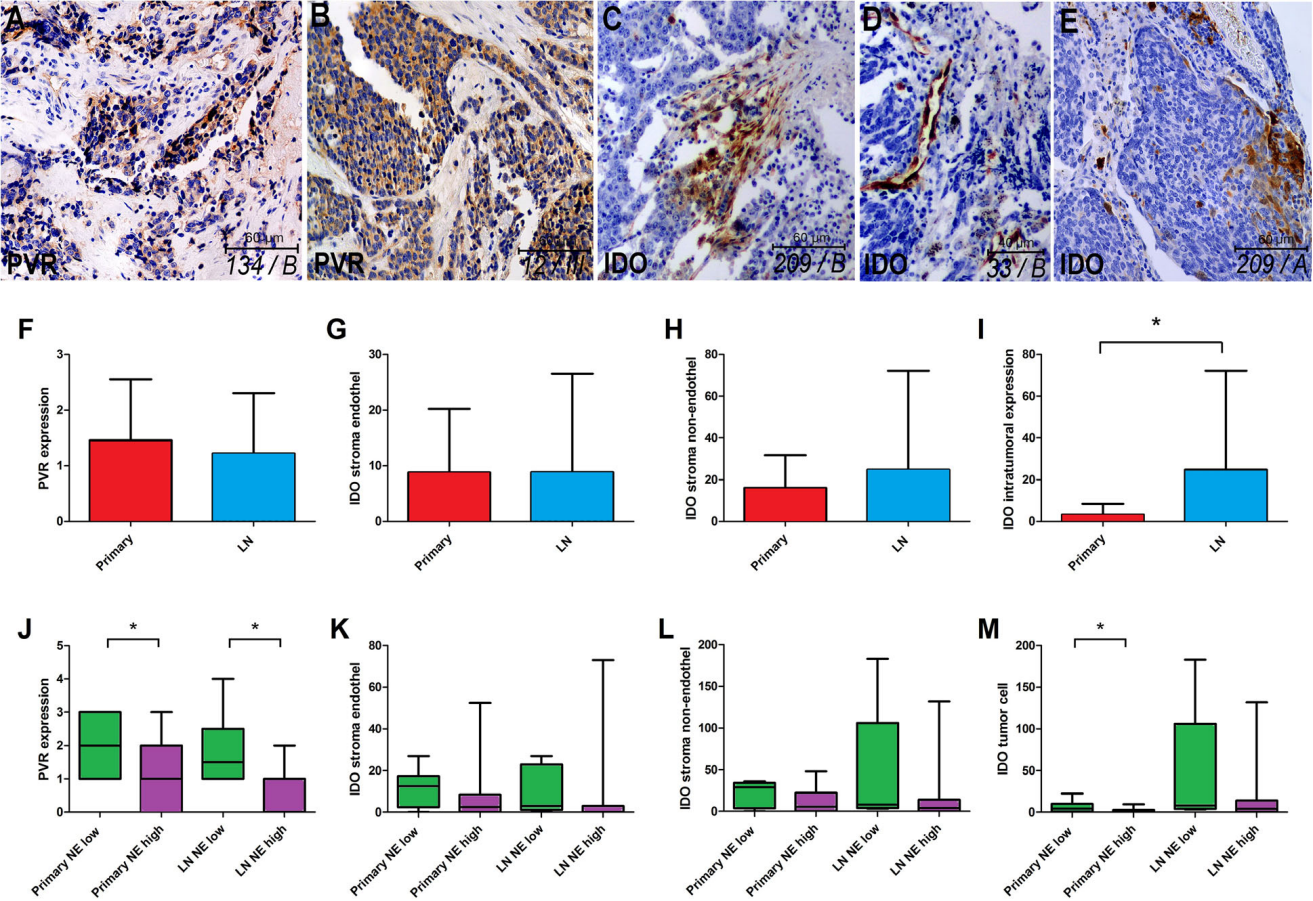
Distribution pattern of immune checkpoint PVR and IDO expression. PVR is expressed by tumor cells, but is not present in stromata including both NE SCLC subtypes (A, B). IDO is expressed by endothelial cells (D), stromal cells of various morphology (C), and by immune cells in tumor nests (E) in both NE SCLC subtypes. PVR expression showed no significant difference in primary tumor vs LN metastases (1.45 ± 0.22 vs 1.21 ± 0.22, *P* = 0.425, *n* = 50, F), but a significantly higher expression in NE‐low vs NE‐high was found both in primary tumors (2.11 ± 0.3 vs 1.03 ± 0.26, *P* = 0.024, *n* = 27, J) and LN metastases (1.83 ± 0.47 vs 0.69 ± 0.2, *P* = 0.032, *n* = 23, J). There were no significant differences between primary tumors and LN metastases regarding the IDO expression in stroma endothelial (8.78 ± 2.09 vs 8.83 ± 3.61, *P* = 0.248, *n* = 48, G) and nonendothelial cells (16.02 ± 3.26 vs 24.79 ± 9.68, *P* = 0.541, *n* = 48, H), whereas the intratumoral expression of IDO was significantly higher in LN metastases compared to primary tumors (3.17 ± 1.09 vs 24.79 ± 9.68, *P* = 0.023, *n* = 47, I). IDO stroma endothelium and nonendothelial cell expression showed no significant difference according to NE‐low and NE‐high tumor subtypes including primary tumors (11.75 ± 3.18 vs 7.56 ± 3.49, *P* = 0.121, *n* = 26, K and 21.81 ± 5.34 vs 12.93 ± 4, *P* = 0.256, *n* = 26, L) and LN metastases (10.20 ± 5.39 vs 8.47 ± 4.4, *P* = 0.196, *n* = 22, K; and 45.60 ± 34.66 vs 19.32 ± 8.55, *P* = 0.172, *n* = 22, L). In contrast, the intratumoral expression of IDO was significantly higher in NE‐low primary tumors (vs NE‐high tumors; 7.31 ± 2.15 vs 1.3 ± 0.65, *P* = 0.041, *n* = 26, M), but not in LN metastases (45.60 ± 34.66 vs 19.32 ± 8.55, *P* = 0.172, *n* = 21, M). Mann–Whitney *U*‐test was used to compare PVR and IDO expression in primary tumors vs LN metastases and in NE‐high vs NE‐low primary tumors and LN metastases. Metric data were shown as mean and corresponding SEM, and graphs indicate the mean and corresponding 95% CI. Statistical significance *P < 0.05.

The expression pattern of emerging immune checkpoints PVR and IDO in primary tumors and LN metastases according to NE‐high vs NE‐low tumors is shown in Fig. 4. IHC shows that PVR is expressed by tumor cells, but not by stromal cells in both NE SCLC subtypes (Fig. 4A,B). IDO is expressed by endothelial cells (Fig. 4D) and stromal cells of various morphology (Fig. 4C), just as by immune cells in tumor nests (Fig. 4E) in both NE SCLC subtypes. PVR expression showed no significant difference in primary tumors vs LN metastases (Fig. 4F). However, in NE‐low subtype, a significantly higher expression was found compared in the NE‐high subtype both in primary tumors (*P* = 0.024) and in LN metastases (*P* = 0.032; Fig. 4J).

There were no significant differences in IDO expression of stroma endothelial (Fig. 4G) and nonendothelial cells (Fig. 4H) between primary tumors and LN metastases. In contrast, intratumoral expression of IDO was higher by orders of magnitude in LN metastases compared to primary tumors (*P* = 0.023; Fig. 4I). We also assessed IDO expression in different NE phenotypes. IDO stroma endothelium and nonendothelial cell expression showed no significant difference between NE‐low and NE‐high tumor subtypes neither in primary tumors nor in LN metastases (Fig. 4K,L). On the contrary, intratumoral expression of IDO was significantly higher in NE‐low (vs NE‐high) primary tumors (*P* = 0.041), but not in LN metastases (Fig. 4M).

**Fig. 5:**
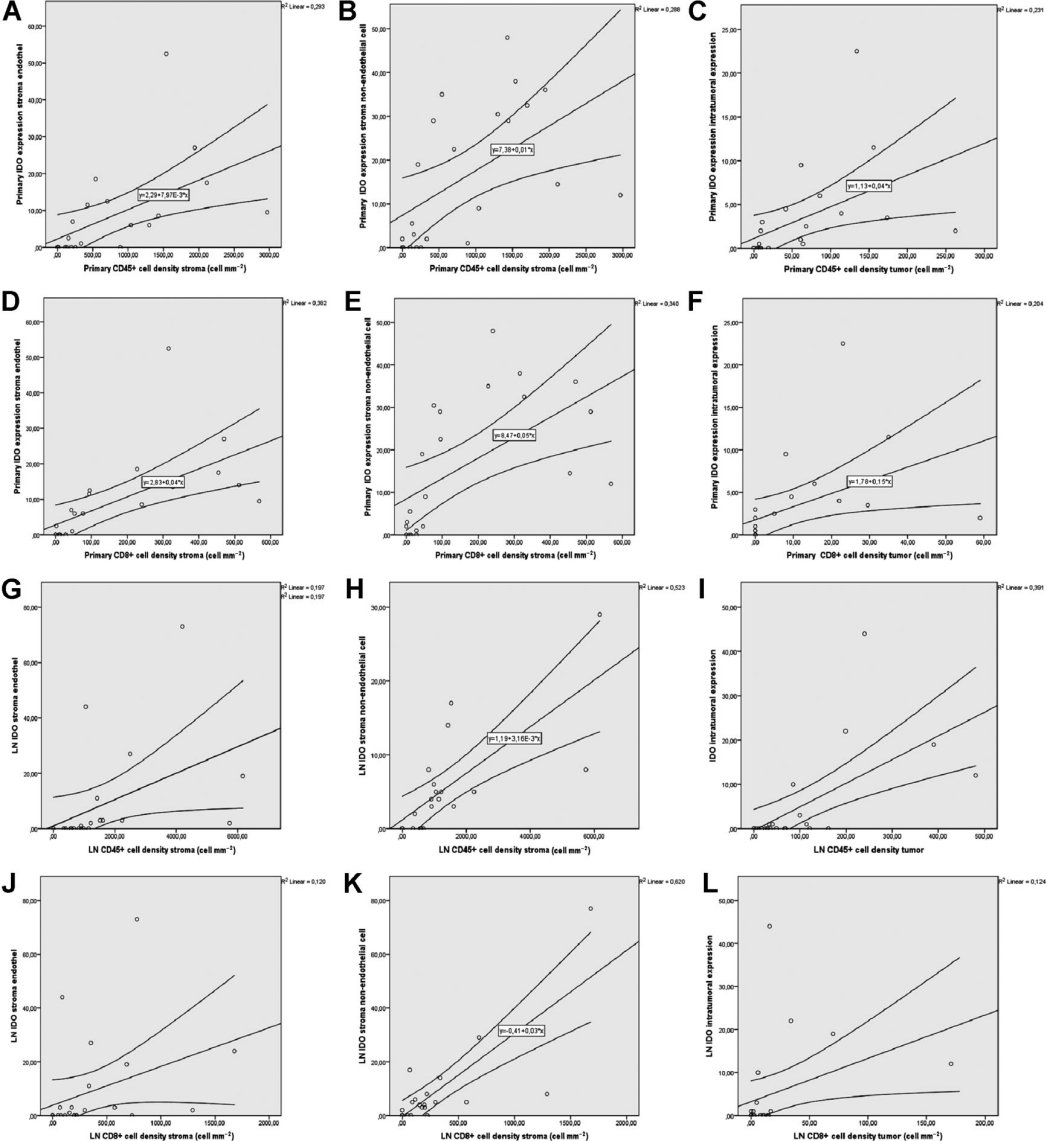
Plot diagrams of significant moderate‐to‐strong correlations between immune checkpoints and immune cell infiltration. There was a significant strong positive correlation between primary tumor IDO stroma endothelium, stroma nonendothelial cell, and immune cell density in stroma including CD45^+^ cells (*r* = 0.78 and *r* = 0.7, respectively, *P* < 0.001, A, B), and CD8^+^ T cells (*r* = 0.87 and *r* = 0.77, respectively, *P* < 0.001, D, E). Similarly, there was a statistically significant strong positive correlation between primary tumor IDO intratumoral expression and immune cell density in tumor nests, including CD45^+^ cells (*r* = 0.79, *P* < 0.001, C), and CD8^+^ T cells (*r* = 0.8, *P* < 0.001, F). A statistically significant strong positive correlation was present between IDO stroma endothelium, stroma nonendothelial cell, and CD45^+^ cell density in the stroma of LN metastases (*r* = 0.8 and *r* = 0.8, respectively, *P* < 0.001, G, H). There was a significant moderate positive correlation between IDO stroma endothelium (*r* = 0.54, *P* < 0.006) and CD8^+^ T‐cell density and a strong positive correlation between stroma nonendothelial cell and CD8^+^ T‐cell density (*r* = 0.68, *P* = 0.001) in the stromata of LN metastases (J, K). Moreover, there was a statistically significant strong positive correlation, between primary tumor IDO intratumoral expression and immune cell density in tumor nests, including CD45^+^ cells (*r* = 0.68, *P* < 0.001, I), and a moderate positive correlation between the same parameters, regarding CD8^+^ T cells (*r* = 0.58, *P* < 0.001, L). Spearman's rank correlation was used for variables CD45, CD3, CD8, IDO, and Kendall's Tau‐b (Kendall rank correlation coefficient) for ordinal variable PVR. Metric data were shown as mean and corresponding SEM, and graphs indicate the mean and corresponding 95% CI.

Next, we investigated the associations between the expression of immune checkpoints and immune cell infiltration (Table 1), where we found a significantly strong positive correlation between IDO stroma endothelium, stroma nonendothelial cell, and immune cell density in stroma including CD45^+^ cells (Fig. 5A,B) and CD8^+^ T cells (Fig. 5D,E) in primary tumors. Furthermore, there was a statistically significant strong positive correlation between primary tumor IDO expression and immune cell density in tumor nests, including CD45^+^ cells (Fig. 5C), and CD8^+^ T cells (Fig. 5F). In terms of LN metastases, we found a statistically significant strong positive correlation between IDO stroma endothelium, stroma nonendothelial cell, and stromal CD45^+^ cell density (Fig. 5G,H) and a significant moderate positive correlation between IDO stroma endothelium, stroma nonendothelial cell, and stromal CD8^+^ T‐cell density (Fig. 5J,K). Moreover, there was a statistically significant strong positive correlation between IDO expression and immune cell density in tumor nests including CD45^+^ cells (Fig. 5I) and CD8^+^ T cells (Fig. 5L). Plot charts of statistically significant correlations between PVR expression and immune cell density are shown in Fig. S3.

**Table 1:** Correlation of immune checkpoints expression and immune cell infiltration. *n*: number of patients; *r*: Correlation Coefficient; *P*: probability value; Primary: primary tumor; Stroma: tumor stroma area; Tumor: tumor nest including intratumoral area.

			Primary tumor cell density (cell·mm^−2^)						LN metastasis cell density (cell·mm^−2^)					
			CD45^+^		CD3^+^		CD8^+^		CD45^+^		CD3^+^		CD8^+^	
			Stroma	Tumor	Stroma	Tumor	Stroma	Tumor	Stroma	Tumor	Stroma	Tumor	Stroma	Tumor
Primary	PVR expression	*r*	0.399^a^	0.529^b^	0.360^a^	0.426^a^	0.406^a^	0.500^b^	0.25	0.12	0.34	0.20	0.30	0.21
		*P*	0.012	0.001	0.024	0.011	0.011	0.004	0.320	0.640	0.159	0.421	0.219	0.413
		*n*	24	24	24	24	24	24	18	18	19	18	19	18
	IDO stroma endothelium	*r*	0.782^b^	0.870^b^	0.851^b^	0.907^b^	0.872^b^	0.797^b^	−0.25	−0.04	−0.07	−0.01	−0.03	0.04
		*P*	0.000	0.000	0.000	0.000	0.000	0.000	0.309	0.865	0.754	0.956	0.904	0.878
		*n*	23	23	23	23	23	23	19	18	20	18	20	18
	IDO stroma nonendothelial cell	*r*	0.702^b^	0.770^b^	0.757^b^	0.790^b^	0.772^b^	0.697^b^	−0.22	−0.06	−0.03	0.01	0.02	0.10
		*P*	0.000	0.000	0.000	0.000	0.000	0.000	0.367	0.817	0.899	0.954	0.919	0.686
		*n*	23	23	23	23	23	23	19	18	20	18	20	18
	IDO tumor	*r*	0.722^b^	0.796^b^	0.767^b^	0.829^b^	0.812^b^	0.801^b^	−0.04	0.24	0.12	0.33	0.16	0.29
		*P*	0.000	0.000	0.000	0.000	0.000	0.000	0.858	0.339	0.606	0.180	0.491	0.252
		*n*	23	23	23	23	23	23	19	18	20	18	20	18
LN	PVR expression	*r*	−0.10	−0.03	−0.12	−0.10	−0.12	−0.25	0.527^b^	0.507^b^	0.355^a^	0.543^b^	0.30	0.521^b^
		*P*	0.619	0.901	0.570	0.638	0.558	0.234	0.002	0.003	0.035	0.002	0.076	0.004
		*n*	25	24	25	24	25	24	22	22	22	22	22	22
	IDO stroma endothelium	*r*	−0.39	−0.08	−0.20	0.09	−0.17	−0.07	0.806^b^	0.641^b^	0.450^a^	0.515^a^	0.542^b^	0.523^a^
		*P*	0.062	0.720	0.341	0.671	0.415	0.762	0.000	0.001	0.031	0.012	0.006	0.010
		*n*	24	23	24	23	24	23	23	23	23	23	23	23
	IDO stroma nonendothelial cell	*r*	−0.22	−0.06	−0.09	0.10	−0.02	0.17	0.801^b^	0.779^b^	0.636^b^	0.665^b^	0.681^b^	0.679^b^
		*P*	0.299	0.772	0.686	0.634	0.914	0.432	0.000	0.000	0.003	0.001	0.000	0.000
		*n*	24	23	24	23	24	23	23	23	23	23	23	23
	IDO tumor	*r*	−0.22	0.01	−0.06	0.19	−0.05	−0.05	0.661^b^	0.689^b^	0.30	0.558^b^	0.40	0.581^b^
		*P*	0.306	0.981	0.776	0.386	0.809	0.815	0.001	0.000	0.171	0.006	0.053	0.004
		*n*	24	23	24	23	24	23	22	23	23	23	23	23

^a^ Correlation is significant at the 0.05 level (two‐tailed Spearman test).

^b^ Correlation is significant at the 0.01 level (two‐tailed Spearman test).

### 3.4 Expressional analysis of MHC II protein and T‐cell exhaustion markers TIM3, PD1, and LAG3

**Fig. 6:**
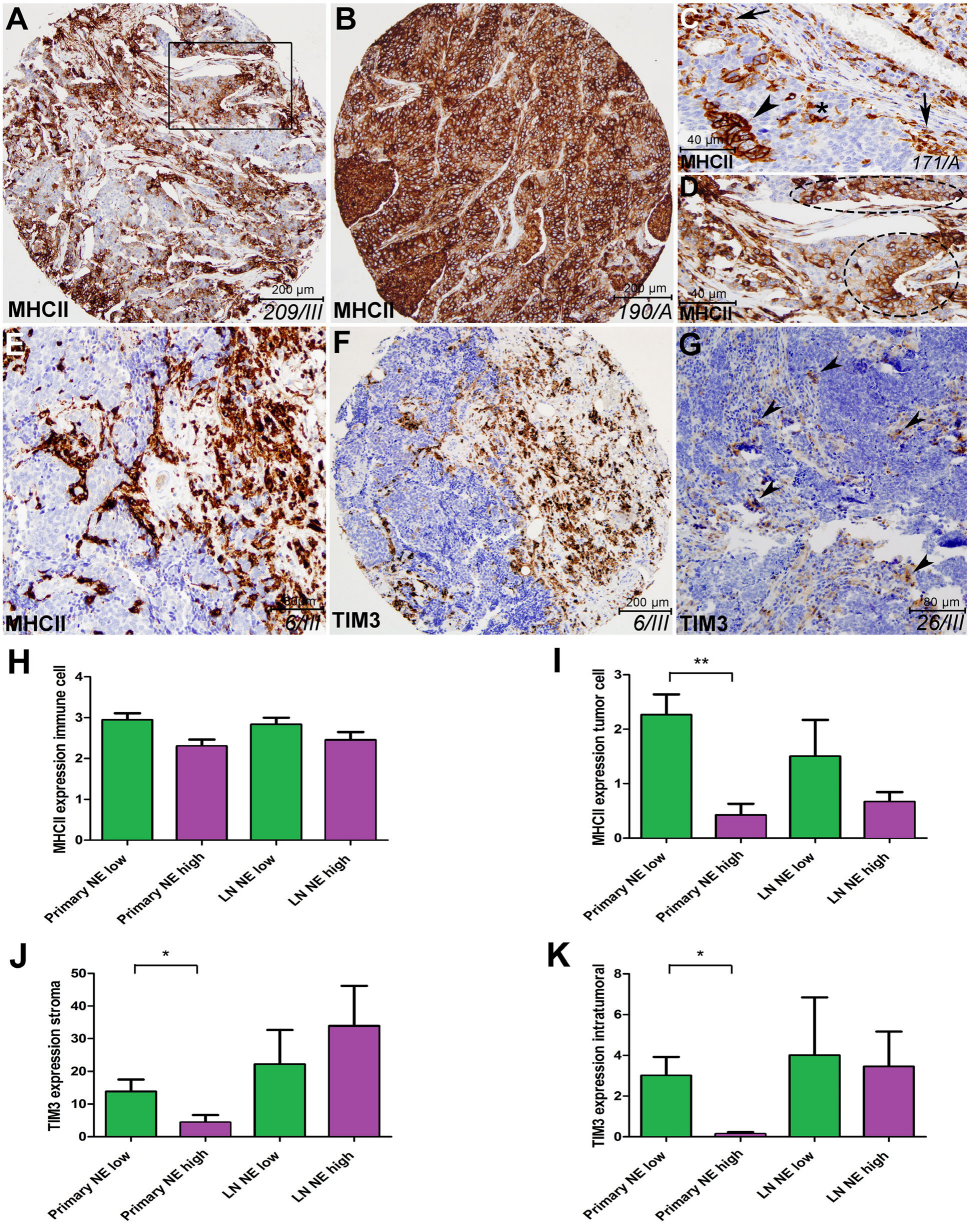
MHC II and lymphocyte exhaustion marker expression in SCLC. Qualitative *in situ* IHC data on representative primary tumor and LN metastasis samples (ID of samples in italics) show strong expression of MHC II protein (A, B). MHC II is expressed in stromal (C arrows), intratumoral immune cells (C asterisk), and in cancer cells of tumor nests similarly (C arrowhead). MHC II staining in tumor nests can be diffuse (B), or mosaical (D encircled area, magnified inset from A). NE‐low tumor shows strong expression of MHC II molecule on cancer cells and on stromal and intratumoral immune cells (A–D), whereas NE‐high tumor exhibits strong stromal MHC II expression on immune cells, but not in tumor nests or cancer cells (E). TIM3 protein is expressed in stromal lymphocytes and scattered immune cells in tumor nests of NE‐low LN metastasis (F). In NE‐high tumors, only a low number of TIM3^+^ cells occur sparsely in stromal brands (G). There were no significant differences in immune cell expression (stromal and intratumoral pooled) of MHC II molecule between NE‐low and NE‐high tumors neither in primary tumors (2.83 ± 0.22 vs 2.29 ± 0.19, *P* = 0.086, *n* = 30, H), nor in LN metastases (2.82 ± 0.16 vs 2.45 ± 0.19, *P* = 0.289, *n* = 27, H). However, tumor cells show significantly stronger MCII expression in NE‐low versus NE‐high primary tumors (2.11 ± 0.45 vs 0.41 ± 0.25, *P* = 0,004, *n* = 30, I), but not in LN metastases (1.5 ± 0.67 vs 0.66 ± 0.18, *P* = 0.298, *n* = 27, I). NE‐low tumors show significantly higher expression of TIM3 compared to NE‐high tumors in the stroma of primary tumors (12.31 ± 4.77 vs 4.07 ± 2.48, *P* = 0.025, *n* = 29, J), but not in LN metastases (22.17 ± 10.53 vs 33.9 ± 12.24, *P* = 0.307, *n* = 27, J). In tumor nests (tumor), expression of TIM3 is significantly higher in NE‐low compared to NE‐high primary tumors (2.87 ± 1.15 vs 0.15 ± 0.1, *P* = 0.015, *n* = 29, K), but the same difference is not significant in LN metastases (4 ± 2.48 vs 3.44 ± 1.72, *P* = 0.905, *n* = 27, K). Mann–Whitney U‐test was used to compare MHC II and TIM3 expression in primary tumors vs LN metastases and in NE‐high vs NE‐low primary tumors and LN metastases. Metric data were shown as mean and corresponding SEM, and graphs indicate the mean and corresponding 95% CI. Statistical significance *P < 0.05; **P < 0.01.

In our analysis, we performed immunostainings and *in situ* expression‐based scoring of MHC II molecule, pivotal in antigen presentation and immunological crosstalk in the tumor microenvironment [27]. Stainings on representative tissue samples show strong MHC II expression in the majority of SCLC tumors. Interestingly, apart from immune cells in the stroma and tumor compartments, MHCII is also expressed on cancer cells of tumor nests, especially in NE‐low tumors (Fig. 6A–E). Of note, in certain samples, tumor cells showed diffuse expression of MHC II (Fig. 6B), in some tumors the molecule occurred exclusively on clusters of cancer cells, scattered in tumor nests (Fig. 6A,C,D). Although there was no significant difference in the immune cell expression of MHC II between NE‐low and NE‐high tumors (Fig. 6H, tumor cells showed significantly higher MHC II expression in NE‐low compared to NE‐high primary tumors (*P* = 0.004; Fig. 6I).

Next, we evaluated the extent of lymphocyte exhaustion in SCLC. We performed IHC with antibodies against TIM3, PD1, and LAG3 molecules. None of the tissue samples of our 32 patients' cohort (neither primary tumors nor LN metastases) displayed positivity for PD1 or LAG3. On the contrary, TIM3 expression was present on lymphocytes of stromal bands and tumor nests as well (Fig. 6E,F). Both stromal expression and intratumoral expression of TIM3 were significantly higher in NE‐low vs NE‐high primary tumors (*P* = 0.025 and *P* = 0.015, respectively), but not in LN metastases (Fig. 6J,K). Of note, the absolute number of TIM3‐positive cells in each sample was considerably higher in LN metastases compared to primary tumors (Fig. 6J,K). PD‐L1 protein expression was not found in any of the samples (data not shown).

## 4 Discussion

The standard of care therapy for extensive‐stage SCLC now includes immunotherapy in the front‐line setting. The addition of atezolizumab or durvalumab to chemotherapy has changed practice recently and is associated with a moderate significantly longer PFS and OS than chemotherapy on its own [1, 2]. However, as of yet, no predictive biomarkers have been identified, and the PFS curves seem to overlap during the initial 8 months, showing that most patients do not benefit from immunotherapy. Additionally, there were increased OS benefits for selected patients that might respond to immunotherapy.

Recent advancements in transcriptomics studies highlight the potential of a distinct microenvironment in SCLC NE subtypes. Understanding the immunology of NE subtypes might affect the clinical outcome and help lay the framework for immunotherapy administration in this devastating cancer [6, 7, 8]. However, to date, studies have been performed exclusively on NSCLC samples. Therefore, our study aims to fill this gap of knowledge with a detailed IHC analysis of immune cell populations on human SCLC tissue samples. We aim to provide an in‐depth intertumor heterogeneity array of IHC staining on primary tumors vs matched LN metastases on immune cell infiltration and immune activation of stroma and epithelial tumor nests in NE‐low and NE‐high tumor phenotypes. Importantly, to our knowledge, this is the first human study delivering *in situ* proteomics data on immune cell populations in LN metastases of SCLC patients.

Our main findings from this study interpret the proteomic profile of the tumor microenvironment to further highlight the relevance of NE‐low vs NE‐high tumor subtypes in the clinical setting. It is also important to note that the presence of lymphatics‐associated genes might influence any transcriptomic study performed on LN metastases. Therefore, our *in situ* proteomic analysis might overcome the limitations above. Others showed that the extent of immunological infiltration in tumor tissue and the expression of immune checkpoints proved to be a reliable marker for response to anti‐PD‐1 immunotherapy and long‐term survival NSCLC [38], and other malignancies, like breast cancer [39], melanoma [40], colorectal carcinoma [41], and prostatic cancer [42] as well. Another group indicated on NSCLC TMA samples that a high number of stromal CD4^+^ and epithelial and stromal CD8^+^ cells were independent positive prognostic markers, and CD8^+^ tumor‐infiltrating lymphocytes (TILs) can stratify immunotherapy‐treated patients of different clinical outcome [43]. Furthermore, a low level of CD8^+^ lymphocyte infiltration in tumor stroma was positively correlated with an augmented incidence of angiolymphatic tumor invasion [38].

In the current study, we first revealed that immune cell infiltration both in primary tumors and in LN metastases is predominant in loosely arranged stromal bands, but not in tumor nests. Even in selected, relatively highly infiltrated tumors, only about 7% in primary and 5% in LN metastasis of CD45^+^ cells and 5% and 6%, respectively, of CD8^+^ T cells are localized in the close microenvironment of tumor cells (Fig. 2). Furthermore, we established that the stroma of LN metastases had significantly higher immune cell density compared to primary tumors; however, this difference was not significant in tumor nests (Fig. 2). Our analyses demonstrated that both stromal and intratumoral CD3^+^/CD45^+^ cell ratio is limited to 27% when pooling both primary tumors and LN metastases (Fig. S2A). This means that TILs make up only about one quarter of all immune cells regardless of their anatomic (macroscopic) localization (primary tumor vs LN). Consequently, a significant fraction of CD45^+^ cells belongs to populations of macrophages, DCs, neutrophils, or other nonspecific immune cells in SCLC. In contrast to CD3^+^/CD45^+^ cell ratios, we found a significant difference in CD8^+^/CD3^+^ cell ratio in stroma vs tumor when both primary and LN metastases were pooled, meaning tumor stroma has a significantly higher ratio of effector T cells compared to tumor nests (Fig. S2B).

The same TMA sets clustered NE‐high and NE‐low SCLC subsets [34] based on the top RNA genes associated with NE differentiation [9, 44, 45]. The latest preclinical studies suggest that, compared to the NE‐high, the NE‐low subtype is more likely to respond to immunotherapy due to its ‘immune oasis’ phenotype, emphasizing the necessity and importance of molecular and *in situ* immunological characterization before the assessment of therapies to this type of recalcitrant cancer [9]. Therefore, we compared *in situ* the quantitative and qualitative extent of the immunological microenvironment of SCLC tumors according to NE‐low and NE‐high subtypes. In line with previously published data, our results confirm that NE‐low tumors are significantly more infiltrated by immune cells, primarily by CD8^+^ effector T cells [9]. Interestingly, in our study, the CD3^+^/CD45^+^ cell ratio was not significantly different in NE‐low relative to NE‐high tumors, suggesting that the T‐cell population is not predominant, neither in stroma nor in tumor nests of NE‐low tumors. In contrast, a substantially higher percentage of CD8‐expressing lymphocytes are present both in NE‐low primary tumors and in LN metastases (vs NE‐high), and the difference is even more considerable in tumor nests (Fig. 3).

Next, in order to identify targets and further understand the immune microenvironment, we analyzed expression of immune checkpoints. PVR (CD155) has been reported to mediate T cell activation *via* CD226, or impede T lymphocytes by binding to TIGIT. PVR overexpression is associated with poor prognosis in melanoma, colorectal, lung, and pancreatic cancers [46, 47, 48, 49]. Our data show that strong PVR expression was significantly more frequent in NE‐low vs NE‐high tumors, both in primary tumors and in LN metastases. Although PVR overexpression was correlated with poor prognosis in multiple studies [48, 49], we found a significant moderate positive correlation between PVR expression and immune cell density in tumor nests including CD45^+^ and CD8^+^ cells.

Another checkpoint, IDO, belonged to the group of anticancer molecules based on its antipathogenic function [50]. Subsequent studies, however, identified tissue macrophages producing high levels of IDO upon interferon‐gamma (IFN‐γ) stimulation inhibiting effector T‐cell proliferation [51]. IDO expression was reported in lung cancer cell lines [52] and *in situ* in 42–43% of NSCLC samples [53, 54]. We found IDO expression on stromal cells of various morphology. Of note, the presence of IDO in stroma endothelial cells is a novel finding in SCLC. Previous studies showed that the endothelial expression of IDO in metastatic kidney cancer promotes response to immunotherapy and is associated with better PFS [54]. In line with other NSCLC studies, we observed scattered IDO immunolabeling only on tumor nest immune cells but not on tumor cells [54]. The role of IDO was demonstrated in other respiratory conditions as well, like pneumonia, where inflammatory macrophages were identified as a primary source of the molecule [56]. IDO expression was not different in stromal cellular elements or endothelium according to NE subtypes. However, IDO expression in tumor nests showed significantly higher levels in NE‐low tumors. Consequently, establishing an immunosuppressive microenvironment for TILs that might explain why NE‐low tumors do not unequivocally have better prognosis despite their ‘immune oasis’ phenotype. Stroma IDO expression might be associated with many types of inhibitory cells in the immunosuppressive tumor microenvironment, like cancer‐associated fibroblasts, myeloid‐derived suppressor cells, or tumor‐associated macrophages, which requires further confirmation. Interestingly, intratumoral expression of IDO showed a conspicuous discrepancy in LN metastases where IDO‐positive cells were much more abundant, than in primary tumors (Fig. 4I). Our findings show that LN metastases are significantly more infiltrated by immune cells (vs primary tumors). This might result in clinically indifferent molecular behavior and aggressiveness of LN metastases due to their distinct immunological microenvironment and immune checkpoint expression patterns. Moreover, there was a statistically significant strong positive correlation between intratumoral expression of IDO and immune cell density in tumor nests including CD45^+^ cells (Fig. 5C) and CD8^+^ T cells (Fig. 5F). Our data suggest that IDO overexpression is an escape mechanism of tumor cells making immune cells and lymphocytes entering tumor nests anergic and unable to launch an immune response against them.

In lung cancer, it was previously shown in cell lines [57] and tissue samples that some tumor cells displayed MHC II expression, mostly in the vicinity of TILs in highly infiltrated tumors [58]. The latter fact suggests that immune cell infiltration may induce MHC II expression in tumor cells in a permissive microenvironment. In our study, we also revealed that MHC II molecules are expressed *in situ* on cancer cells of certain SCLC tumors, but predominantly in the NE‐low subtype (Fig. 6), whereas PD‐1 and PD‐L1 protein expression was not detectable *in situ* in any of the samples. This finding is similar to other researchers' that reported a relatively low rate of PD‐L1 expression in SCLC up to 35.0% (with a very low cutoff point of 5% for PD‐L1 positive/negative expression), which was consistently lower than that in NSCLC [59]. This difference can be explained by a variety of factors, including tumor stage and assays used. We found no expression of lymphocyte exhaustion marker LAG3 in our study showing that LAG3 is not relevant in the SCLC microenvironment. In contrast, TIM3 was expressed by significantly more TILs in NE‐low compared to NE‐high tumors. Irrespective of NE subtypes, TIM3 was more expressed in LN metastases compared to primary tumors. In connection with the clinical relevance of TIM3, recently it was reported that inhibition of the TIM3‐related molecular pathway promoted anticancer immunity and increased IFN‐γ production of T cells [60]. Immune checkpoints PD‐1, TIM‐3, and LAG‐3 were also shown to be upregulated in TILs of hepatocellular carcinomas and may enhance T‐cell response to tumor antigens in a synergistic way [61]. Altogether, our results confirm that NE‐low tumors and LN metastases (regardless of NE phenotype) seem to be more immunogenic, with higher immune checkpoint and lymphocyte exhaustion molecule expression.

Limitations of this study include that it is a retrospective cross‐sectional study with limited clinicopathological data available. The patient population is unique in terms of the resected sample size and corresponding LN availability; however, it is small even in the light of the fact that matched tumor samples are usually not available in the case of SCLC. Prognostic data are limited, and our study may be influenced by the differences in the administration of oncotherapy including surgical techniques over a long period.

## 5 Conclusion

To our knowledge, this is the first human study that demonstrates *in situ* that SCLC stroma is more infiltrated by immune cells compared to tumor nests. Additionally, NE‐low tumors are more infiltrated by immune cells compared to NE‐high tumors. Therefore, our results suggest that SCLC is classified into two distinct NE subtypes that may alter treatment outcomes. Accordingly, we hypothesize that NE‐low tumors have a microenvironment potentially associated with increased benefit from immune checkpoint inhibitor therapy. Consequently, patient NE subtype should be identified in future clinical trials to select patients that will most likely benefit from immunotherapy administration. Moreover, PVR, IDO, MHCII, and TIM3 are potential new targets in SCLC with increased expression in NE‐low subtype, providing critical insight for further prospective studies on SCLC immunotherapies.

## Conflict of interest

The authors declare no conflict of interest.

## Author contributions

ZL, DD, and BD conceived and designed the study. KS, TH, JM, SR, and SLP made administrative support. ZL, ZM, JM, and BD involved in provision of study materials or patients. DD, ZL, HY, CR, and SLP involved in collection and assembly of data. DD, ZL, CR, and HY made data analysis and interpretation. All authors wrote the manuscript. All authors approved the final manuscript.

## Supporting information


**Fig. S1.** Tumor nest and stroma area ratio in primary SCLC tumors and matched LN metastases, according to NE tumor subtypes. There were no significant differences in stroma and tumor nest (tumor) area ratio in primary tumors versus matched LN metastases (0.84 ± 0.23 vs 0.68 ± 0.24, respectively, *P* = 0.22, *n* = 59 A). No significant differences were present in stroma and tumor area ratio in primary tumors and LN metastases according to NE subtypes (primary NE‐low vs high: 0.62 ± 0.14 vs 1.715 ± 0.85, *P* = 0.92, *n* = 31; LN NE‐low vs high: 0.6915 ± 0.25 vs 0.6878 ± 0.3, respectively, *P* = 0.377, *n* = 28, B).

**Fig. S2.** Relative distribution of immune cells according to primary SCLC tumors and matched LN metastases. There was no significant difference in CD3^+^/CD45^+^ cell ratio between stroma and tumor nests when pooling both primary and LN metastases (27.21 ± 2.02 vs 26.06 ± 2.85, respectively, *P* = 0.73, A), but there was a significant difference in the case of CD8^+^/CD3^+^ cell ratio (56.44 ± 2.78 vs 45.76 ± 4.91, *P* = 0.044, B). There was no significant difference in stromal CD3^+^/CD45^+^ cell ratio according to primary tumors and LN metastases (24.15 ± 2.86, vs 30.35 ± 3.08, *P* = 0.147, C). We found no statistically significant difference in CD3^+^/CD45^+^ cell ratio in tumor nests according to primary tumors and LN metastases (21.26 ± 3.3 vs 29.9 ± 4.87, *P* = 0.146, D). There was no significant difference in CD8^+^/CD3^+^ ratio in stroma according to primary tumors and LN metastases (58.88 ± 3.98, vs 53.43 ± 4.37, *P* = 0.361, E). Similarly, no significant difference was identified in CD8^+^/CD3^+^ ratio in tumor nests according to primary tumors and LN metastases (47.18 ± 8.78 vs 45.14 ± 6.57, *P* = 0.854, F).

**Fig. S3.** Plot diagrams of significant moderate‐to‐strong correlations between PVR and immune cell densities in tumor nests. There were a statistically significant moderate positive correlation between primary tumor PVR expression and CD45^+^ (*r* = 0.52, *P* = 0.001) and CD8^+^ (*r* = 0.5, *P* = 0.004) immune cell densities in tumor nests (A and B). Furthermore, in terms of LN metastases, a similarly moderate significant positive correlation was found between PVR expression and immune cell densities in tumor nests, including CD45^+^ (*r* = 0.507, *P* < 0.003), CD8^+^ cells (*r* = 0.521, *P* < 0.004, C and D).

**Table S1.** Shows the summary of key protein expression data in the tumor microenvironment, according to NE‐low vs NE‐high SCLC subtypes. Most important mean‐, SEM‐ and *P*‐values.
