# Supplementary figures and images for: Neuroendocrine subtypes of small cell lung cancer differ in terms of immune microenvironment and checkpoint molecule distribution
Source: Mol Oncol. 2020 Jul 18;14(9):1947–65. doi: 10.1002/1878-0261.12741 (PMC7463307; doi:10.1002/1878-0261.12741)

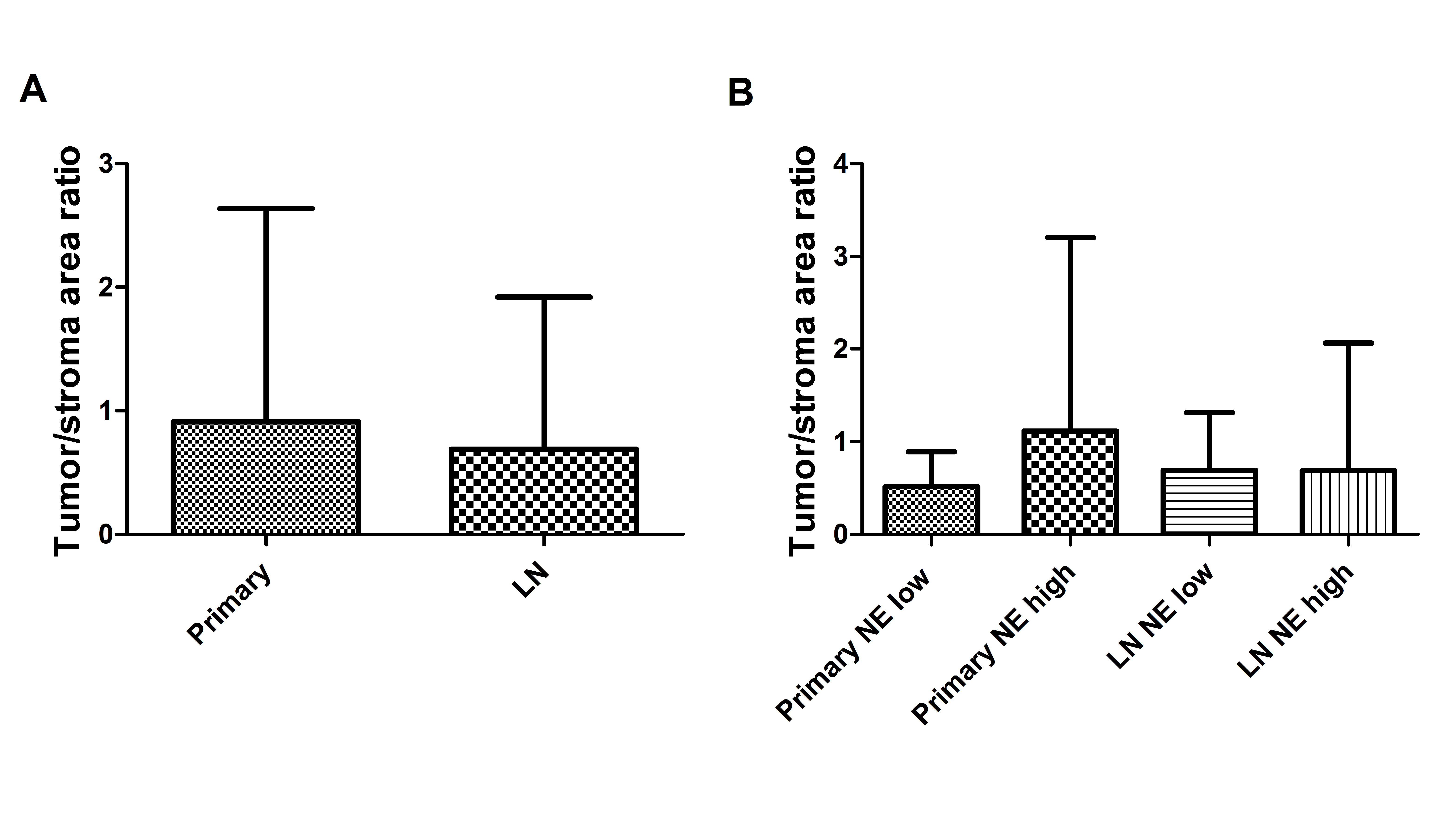

Supplement: Supplementary file 1 — Fig. S1. Tumor nest and stroma area ratio in primary SCLC tumors and matched LN metastases, according to NE tumor subtypes. There were no significant differences in stroma and tumor nest (tumor) area ratio in primary tumors versus matched LN metastases (0.84 ± 0.23 vs 0.68 ± 0.24, respectively, P = 0.22, n = 59 A). No significant differences were present in stroma and tumor area ratio in primary tumors and LN metastases according to NE subtypes (primary NE‐low vs high: 0.62 ± 0.14 vs 1.715 ± 0.85, P = 0.92, n = 31; LN NE‐low vs high: 0.6915 ± 0.25 vs 0.6878 ± 0.3, respectively, P = 0.377, n = 28, B). [file MOL2-14-1947-s001.jpg]

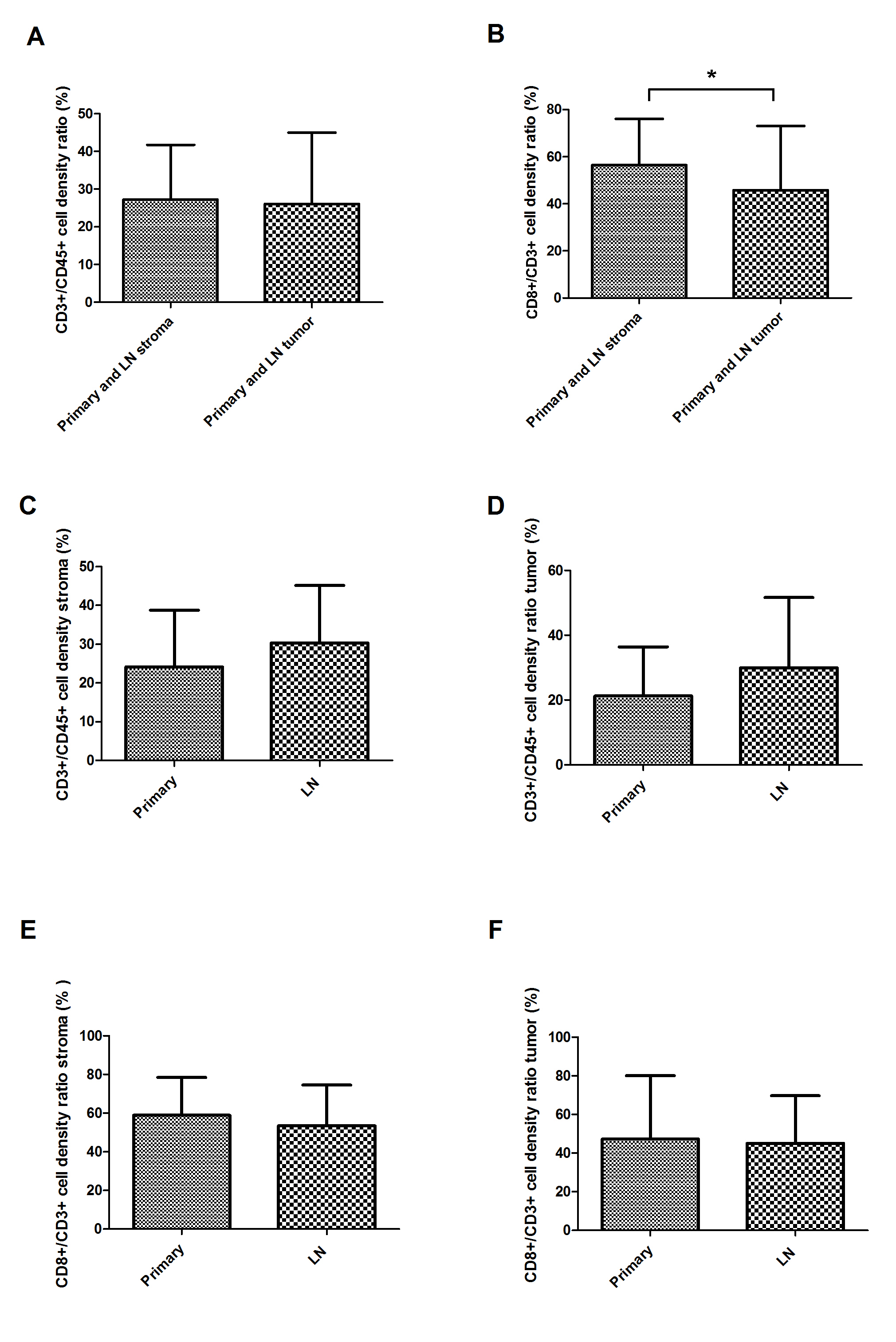

Supplement: Supplementary file 2 — Fig. S2. Relative distribution of immune cells according to primary SCLC tumors and matched LN metastases. There was no significant difference in CD3+/CD45+ cell ratio between stroma and tumor nests when pooling both primary and LN metastases (27.21 ± 2.02 vs 26.06 ± 2.85, respectively, P = 0.73, A), but there was a significant difference in the case of CD8+/CD3+ cell ratio (56.44 ± 2.78 vs 45.76 ± 4.91, P = 0.044, B). There was no significant difference in stromal CD3+/CD45+ cell ratio according to primary tumors and LN metastases (24.15 ± 2.86, vs 30.35 ± 3.08, P = 0.147, C). We found no statistically significant difference in CD3+/CD45+ cell ratio in tumor nests according to primary tumors and LN metastases (21.26 ± 3.3 vs 29.9 ± 4.87, P = 0.146, D). There was no significant difference in CD8+/CD3+ ratio in stroma according to primary tumors and LN metastases (58.88 ± 3.98, vs 53.43 ± 4.37, P = 0.361, E). Similarly, no significant difference was identified in CD8+/CD3+ ratio in tumor nests according to primary tumors and LN metastases (47.18 ± 8.78 vs 45.14 ± 6.57, P = 0.854, F). [file MOL2-14-1947-s002.jpg]

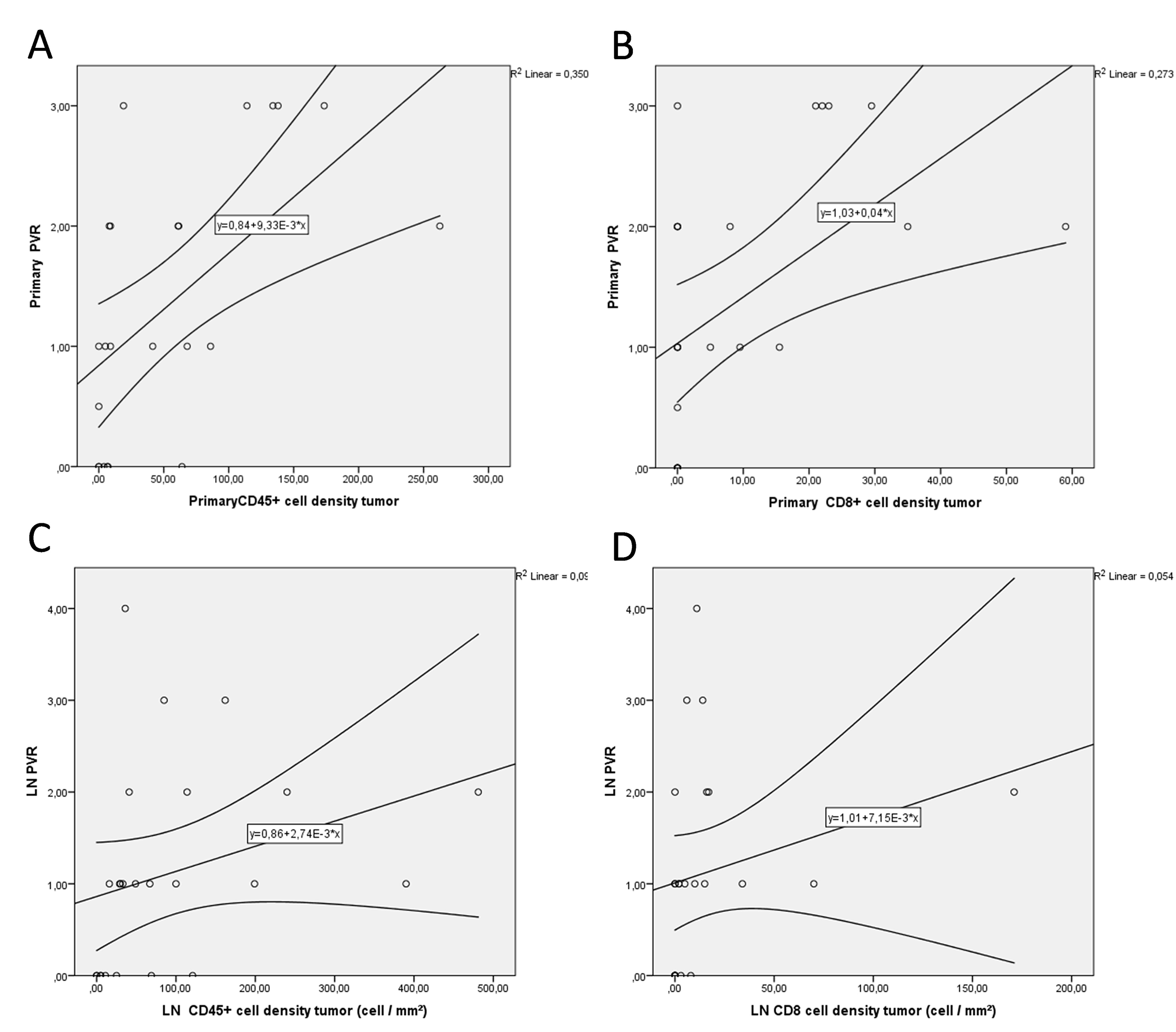

Supplement: Supplementary file 3 — Fig. S3. Plot diagrams of significant moderate‐to‐strong correlations between PVR and immune cell densities in tumor nests. There were a statistically significant moderate positive correlation between primary tumor PVR expression and CD45+ (r = 0.52, P = 0.001) and CD8+ (r = 0.5, P = 0.004) immune cell densities in tumor nests (A and B). Furthermore, in terms of LN metastases, a similarly moderate significant positive correlation was found between PVR expression and immune cell densities in tumor nests, including CD45+ (r = 0.507, P < 0.003), CD8+ cells (r = 0.521, P < 0.004, C and D). [file MOL2-14-1947-s003.jpg]

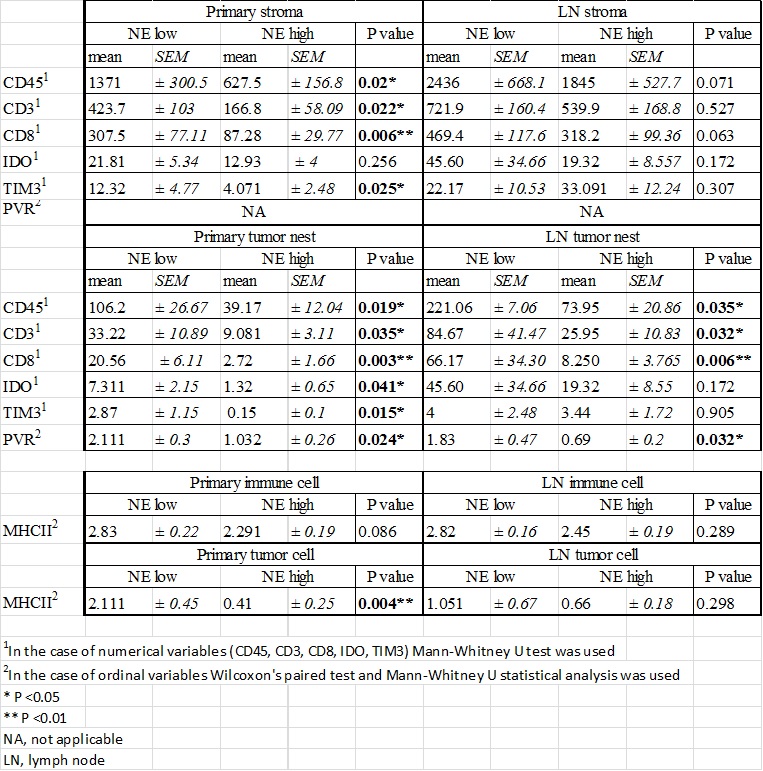

Supplement: Supplementary file 4 — Table S1. Shows the summary of key protein expression data in the tumor microenvironment, according to NE‐low vs NE‐high SCLC subtypes. Most important mean‐, SEM‐ and P‐values. [file MOL2-14-1947-s004.jpg]
